# ﻿Eriolaenabacgiangensis (Malvaceae), a new species from Vietnam

**DOI:** 10.3897/phytokeys.256.152884

**Published:** 2025-05-02

**Authors:** Van Tien Tran, The Bach Tran, Ha Phuong Vu, Hoang Tuan Cao, Thu Ha Bui

**Affiliations:** 1 Department of Natural Resources and Environmental Management, Academy of Public Administration and Governance, 77 Nguyen Chi Thanh Road, Lang Thuong, Dong Da, Hanoi, Vietnam Academy of Public Administration and Governance Hanoi Vietnam; 2 Graduate University of Science and Technology, Vietnam Academy of Science and Technology, 18 Hoang Quoc Viet, Cau Giay, Ha Noi, Vietnam Graduate University of Science and Technology Ha Noi Vietnam; 3 Department of Botany, Institute of Biology, Vietnam Academy of Science and Technology, 18 Hoang Quoc Viet, Cau Giay, Ha Noi, Vietnam Vietnam Academy of Science and Technology Ha Noi Vietnam; 4 Faculty of Biology, Hanoi National University of Education, 136, Xuan Thuy, Cau Giay, Ha Noi, Vietnam Hanoi National University of Education Ha Noi Vietnam

**Keywords:** Bac Giang, Dombeyoideae, *Eriolaena*, Vietnam

## Abstract

The new species *Eriolaenabacgiangensis* from Vietnam is described and illustrated. The identified key was constructed to indicate the differences amongst three allied species of *Eriolaena*. *E.bacgiangensis* was compared with similar species, *E.candollei* and *E.wallichii*. *E.bacgiangensis* differs from *E.candollei* by the length of pedicel (1.4–2.3 cm long vs. 3.0–4.0 cm in *E.candollei*); presence of epicalyx after flowers at anthesis in *E.bacgiangensis* (vs. absence of epicalyx after flowers at anthesis in *E.candollei*); densely fringed epicalyx in *E.bacgiangensis* (vs. sparsely fringed epicalyx in *E.candollei*); style significantly exceeds staminal tube length in *E.bacgiangensis*, while the style is slightly longer than the staminal tube in *E.candollei*; the fruit apex round in *E.bacgiangensis* (vs. pointed and beaked in *E.candollei*). The linear epicalyx lobes are a key diagnostic trait for distinguishing *E.bacgiangensis* from *E.wallichii* (linear vs. ovate in *E.wallichii*). In addition, the other different characteristics of the two species are: leaf blade (not thickly papery in *E.bacgiangensis* vs. thickly papery in *E.wallichii*); length of inflorescences (13.0–18.0 cm in *E.bacgiangensis* vs. ≤ 6.0 cm in *E.wallichii*); shape of flower bud (lanceolate in *E.bacgiangensis* vs. globular in *E.wallichii*); fruit apex (round in *E.bacgiangensis* vs. apex shortly beaked in *E.wallichii*).

## ﻿Introduction

*Eriolaena* DC. was established in 1823. This genus is now recognised as a genus in the subfamily Dombeyoideae, family Malvaceae (Colli-Silva et al. 2025). The genus comprises approximately 17 species (Ya et al. 2007), mainly distributed in tropical and subtropical Asia. In Vietnam, one species of *Eriolaena* (*E.candollei*) has been recorded so far (Gagnepain 1910; Nguyen et al. 1980; Pham 1999; Chamlong 2001; Nguyen 2003; Newman et al. 2007; Ya et al. 2007; Chandramohan et al. 2020; POWO 2025). *E.candollei* includes three synonyms such as *E.affinis* Pierre, *E.glabrescens* DC. and *E.kwangsiensis* Hand.-Mazz. (POWO 2025). The species is native to Bangladesh, Cambodia, south-central China, southeast China, East Himalaya, India, Laos, Myanmar, Thailand and Vietnam (POWO 2025). In 2025, during a botanical survey of Bac Giang Province, Vietnam, specimens of the genus *Eriolaena* were collected from this Province. After comparing with specimens in the Herbaria HN, K, KRIB, P and VNM and consulting the relevant literature, we determined that our specimens represent a new species. Here, we describe and illustrate this new species as *Eriolaenabacgiangensis* T.H.Bui.

## ﻿Materials and methods

All morphological characters of the new species were observed from living and dried specimens. Material was stored at HN Herbarium of the Institute of Biology in Vietnam. The conservation status of the new species was assessed according to the guidelines of the International Union for Conservation of Nature (IUCN 2025). Other specimens of *Eriolaena* species are studied at HN, KRIB, VNM Herbaria that preserve many specimens of species distributed in Vietnam (acronyms follow Thiers (2025)).

## ﻿Taxonomy

### ﻿ Eriolaena bacgiangensis

Taxon classificationPlantae

T.H.Bui sp. nov.

B680676E-A003-5F1F-BA05-58D5CF7623EA

urn:lsid:ipni.org:names:77360921-1

Figs 1 , 2 , 3

#### Type.

Vietnam. • Bac Giang Province, Son Dong District, Huu San Commune, 21°23'26.1"N, 106°57'27.6"E, alt. 178 m, 18 July 2015, *T.B. Tran, D.B. Tran, T.C. Vu, V.H. Do, H.Q. Bui, H.S. Doan*, *VK 6489* (holotype: HN80497!; isotypes: HN80498!, HN80499!, HN80500!, VNM00074095!).

#### Diagnosis.

*Eriolaenabacgiangensis* is most similar to *E.candollei* due to the shape of the leaf blade, number of basal and lateral veins, presence of epicalyx in the flower bud, lanceolate epicalyx; shape of sepal (linear–lanceolate) and yellow petals. *E.bacgiangensis* differs from *E.candollei* by the pedicel length (1.4–2.3 cm), presence of epicalyx after flowers at anthesis, densely fringed epicalyx, style significantly exceeds staminal tube length and fruit apex round.

#### Description.

***Small tree*** up to 8 m tall; branchlets stellate puberulent. Stipules caducous. Petioles 1.1–6.0 cm long, densely pubescent. ***Leaf*** blades cordate, 7.0–23 × 3.6–18.0 cm; apex acute to caudate; base shallowly cordate; surfaces pubescent; veins densely pubescent; stellate hairy, denser abaxially; margin obtusely dentate; basal veins 5–7; secondary veins 3–5-paired. ***Inflorescence*** racemose, axillary to terminal, 3–10-flowered, 13.0–18.0 cm long. Flower pedicels 1.4–2.3 cm long, densely pubescent. ***Flower*** buds with epicalyx; epicalyx horizontal or pendulous; lanceolate, 1.5–1.6 × 0.6–0.7 cm, densely pubescent. ***Epicalyx*** present after flowers at anthesis, 1.3–1.5 × 0.2–0.4 cm; epicalyx lobe linear, margin pinnately partite, densely fringed, densely woolly stellate pilose. Calyx and corolla 5-merous. ***Sepals*** 5, valvate, linear–lanceolate, 2.0–2.9 × 0.47–0.53 cm; abaxially stellate hairy, adaxially villous. ***Petals*** 5, yellow to orange, oblong-rectangle; petals are turned backwards between the sepals; 1.3–1.5 × 0.6–0.7 cm, broader than sepals; above part glabrous; apex entire to emarginate; narrowed towards the base, claw broad, thick densely pubescent, deflexed between the sepals. ***Stamens*** many, connate into cylindrical tube; staminal tube covering the ovary and style, 1.1–1.3 cm long; anthers linear-oblong to rectangular, 0.21–0.31 × 0.04 cm, 2-celled, cells parallel. ***Ovary*** superior, ovate, 0.07–0.08 × 0.05–0.06 cm, pubescent; style linear, simple, 2.0–2.2 cm long, style significantly exceeds staminal tube length; stigma 5–7-lobed, lobes needle-like, horizontal to down-curved. ***Capsules*** ovate-elliptic, woody, 2.0–2.5 × 1.3–1.8 cm, longitudinally grooved, fruit apex round; fruit stalk 3.5–3.7 cm long; densely hairy.

**Figure 1.**
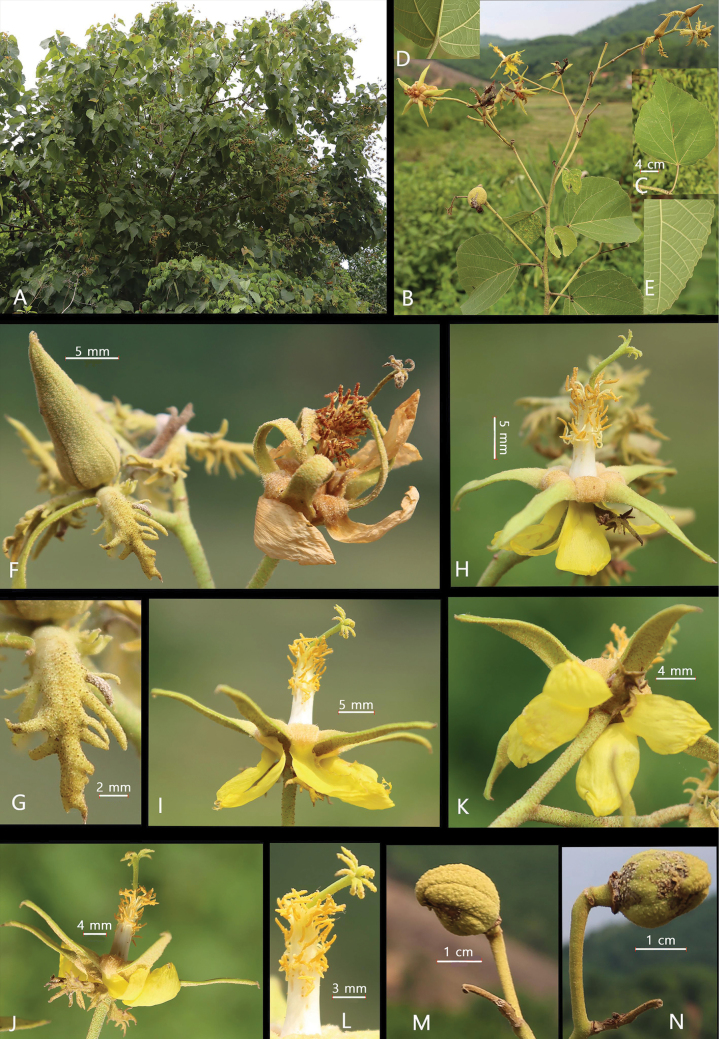
*Eriolaenabacgiangensis* T.H.Bui **A** habit **B** flowering, fruiting branch **C** leaves **D** cordate base **E** dentate margin **F** flower bud and flower **G** epicalyx lobes **H–J** flower **K** flower from below **L** staminal tube, style, stigma **M, N** fruit (Photos by T.B. Tran).

#### Etymology.

The specific epithet refers to the type locality, Bac Giang Province in Vietnam.

#### Distribution and ecology.

*E.bacgiangensis* is found only in Vietnam, Bac Giang Province, Son Dong District, Huu San Commune, where it grows in well-lit places in shrubland and dry soil of secondary forest, in association with *Acaciamangium* Willd., *Bidenspilosa* L., *Cayratiatrifolia* (L.) Mabb. & J.Wen, *Chromolaenaodorata* (L.) R.M.King & H.Rob. and *Stachytarphetajamaicensis* (L.) Vahl. Flowering and fruiting specimens were collected in July 2015.

**Figure 2.**
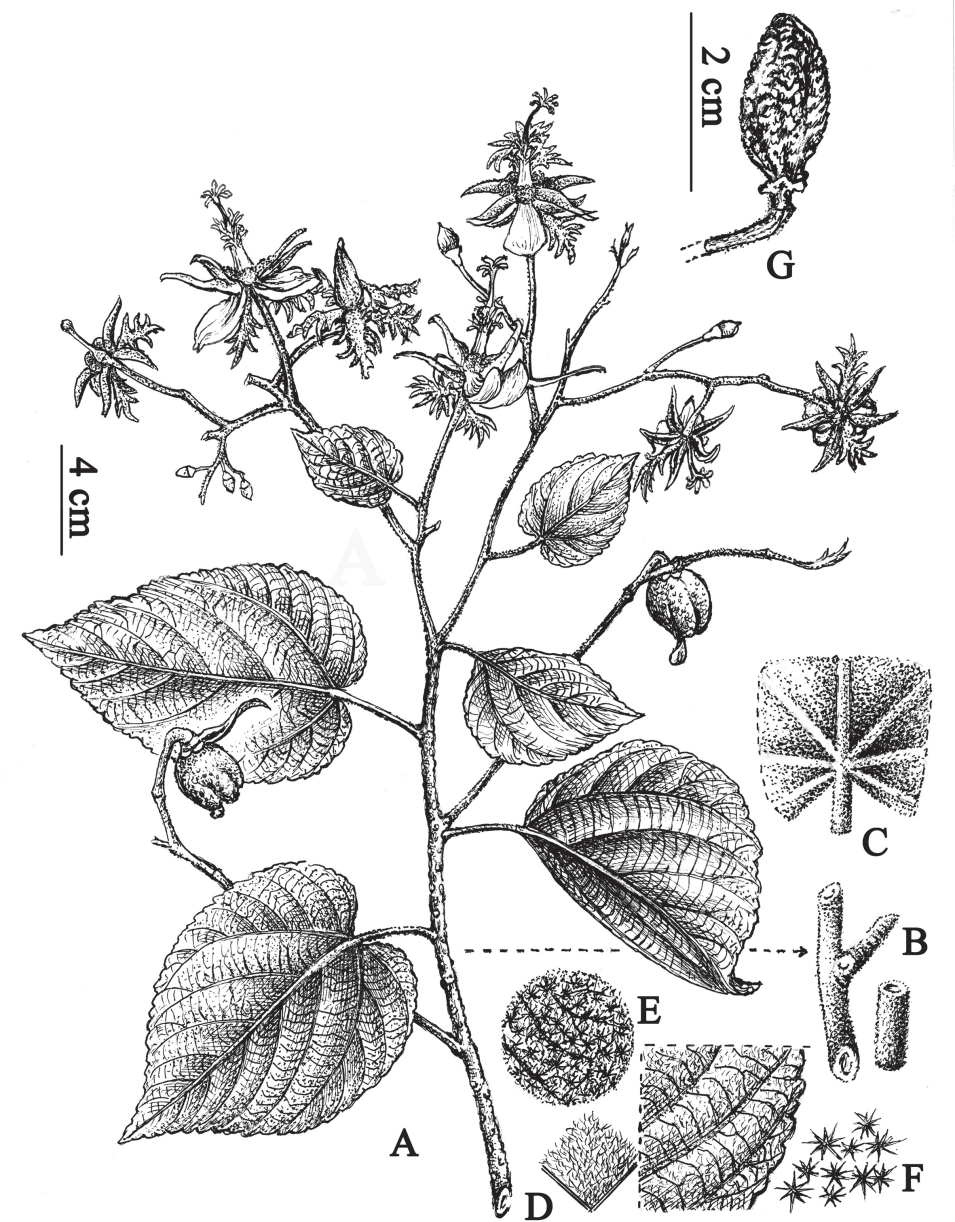
*Eriolaenabacgiangensis* T.H.Bui **A** flowering, fruiting branch **B** pubescent branch **C** pubescent leave **D–F** stellate hairs on leaves **G** fruit (Drawn by Le Kim Chi).

#### Conservation status.

Data Deficient (DD) (IUCN 2025). *Eriolaenabacgiangensis* is known only from the type locality. A comprehensive botanical survey of the *Eriolaena* has not been carried out to date. Potential threats from habitat fragmentation for road construction and land-use change for *Acacia* spp. plantations, therefore, require urgent and appropriate plans for ex situ species conservation.

**Figure 3.**
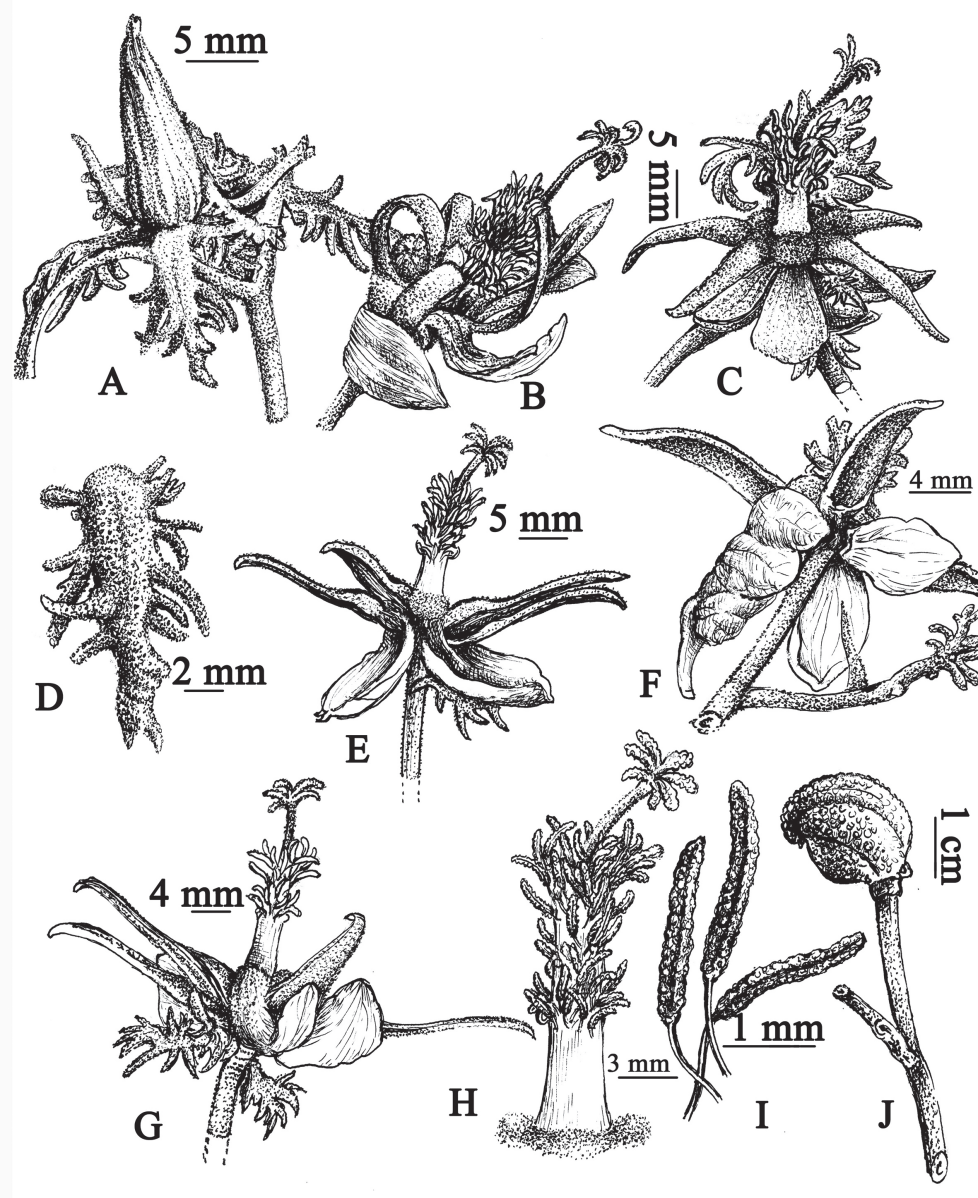
*Eriolaenabacgiangensis* T.H.Bui **A** flower bud **B, C** bloom flower **D** epicalyx lobes **E, G** flower **F** flower from below **H** staminal tube, style, stigma **I** stamens **J** fruit (Drawn by Le Kim Chi).

## ﻿Discussion

Diagnostic characters separating the allied species are listed in Table 1. Based on keys to species of *Eriolaena* mentioned in previous works (Ya et al. 2007; Chandramohan et al. 2020), we determined two species of *Eriolaena* (*E.candollei*, *E.wallichii*) with some similar features to the specimens we collected (branchlets stellate pubescent, stipules caducous, inflorescence axillary or terminal, pedicel with stellate hairy, flower bud with epicalyx, sepals 5, sepal linear–lanceolate, petals 5, fruit pubescent).

**Table 1.** Morphological comparison of *E.bacgiangensis* with similar species.

Characters	*E.bacgiangensis*	*E.candollei*	*E.wallichii*
**Habit**	small tree up to 8 m tall	tree up to 20 m tall	trees up to 6 m tall
**Shape of flower bud**	lanceolate	lanceolate	globular
**Epicalyx (absent/present) after flowers at anthesis**	present	absent	present
**Margins of epicalyx lobes**	deeply, densely fringed	sparsely fringed	deeply fimbriate
**Comparison between style and staminal tube**	style significantly exceeds staminal tube length	style slightly longer than staminal tube	–
**Fruit apex**	round	pointed and beaked	apex shortly beaked
**Length of fruit stalk (cm)**	3.5–3.7	1.0–3.0	ca. 1.5

Two easily recognisable features of the epicalyx which we used to distinguish *E.bacgiangensis* from *E.candollei* include the presence of the epicalyx after flowers at anthesis and densely fringed epicalyx in *E.bacgiangensis* (vs. absence of epicalyx after flowers at anthesis and sparsely fringed epicalyx in *E.candollei*).

The shape of the epicalyx lobe can be used to distinguish *E.bacgiangensis* from *E.wallichii* (linear vs. ovate in *E.wallichii*). Therefore, the characteristics of the epicalyx are very important for the identification of *E.bacgiangensis.* In addition, the other differences in characteristics of the two species are: leaf blade (not thickly papery in *E.bacgiangensis* vs. thickly papery in *E.wallichii*); length of inflorescences (13.0–18.0 cm in *E.bacgiangensis* vs. ≤ 6.0 cm in *E.wallichii*); shape of flower bud (lanceolate in *E.bacgiangensis* vs. globular in *E.wallichii*); fruit apex (round in *E.bacgiangensis* vs. apex shortly beaked in *E.wallichii*).

### ﻿Key to three species *E.bacgiangensis*, *E.candollei* and *E.wallichii*

**Table:** 

1	Inflorescences ≤ 6.0 cm long. Flower bud globular	***E.wallichii***
–	Inflorescences ≥ 7.0 cm long. Flower bud lanceolate	**2**
2	Pedicel 3.0–4.0 cm long. Epicalyx lobes sparsely fringed, epicalyx absent after anthesis. Width of ovary 0.2–0.3 cm long, style slightly longer than staminal tube. Apex of fruit pointed and beaked, fruit stalk 1.0–3.0 cm long	***E.candollei***
–	Pedicel 1.4–2.3 cm long. Epicalyx lobes densely fringed, epicalyx present after anthesis. Width of ovary 0.5–0.6 cm long, style much longer than the staminal tube. Apex of fruit round, fruit stalk 3.5–3.7 cm long	***E.bacgiangensis***

### ﻿Additional specimens examined

*E.candollei*: VIETNAM. Lang Son: Lang Met, 4/7/1944, Petelot, Petelot 6899 (VNM); Ninh Binh: Cuc Phuong, N.M Cuong, NMC 139 (HN); Thanh Hoa, 1921, Poilane, Poilane 1611 (VNM); Kon Tum: Sa Thay, Sa Son, 4/5/2009, T.T. Bach, V.T Chinh, D.V. Hai, B.H. Quang, VK 2630 (HN, KRIB); Dak Lak: Buon Ma Thuot, 17/1/1956, Pierre, Pierre 133 (VNM). LAOS: Xiangkhouang, 6/4/1949, Vidal, s.n. (VNM).

## Supplementary Material

XML Treatment for Eriolaena bacgiangensis

## ﻿Additional information

### Author ORCIDs

Van Tien Tran https://orcid.org/0000-0001-7407-318X

The Bach Tran https://orcid.org/0000-0003-3798-5969

Ha Phuong Vu https://orcid.org/0009-0009-5765-5343

Hoang Tuan Cao https://orcid.org/0009-0002-3487-8295

Thu Ha Bui https://orcid.org/0000-0001-6971-0105

### Data availability

All of the data that support the findings of this study are available in the main text.
